# Heightened expression of MICA enhances the cytotoxicity of NK cells or CD8^+^T cells to human corneal epithelium in vitro

**DOI:** 10.1186/1471-2415-12-6

**Published:** 2012-04-04

**Authors:** Jiaxu Hong, Ting Qiu, Tingting Qian, Gang Li, Xiaobo Yu, Junyi Chen, Qihua Le, Xinghuai Sun, Jianjiang Xu

**Affiliations:** 1 Department of Ophthalmology, Eye, Ear, Nose, and Throat Hospital, School of Shanghai Medicine, Fudan University, 83 Fenyang Road, Shanghai 200031, China; 2 Shanghai Children’s Medical Center, Jiaotong University, 1678 Dongfeng Road, Shanghai 200127, China; 3 State Key Laboratory of Medical Neurobiology, Institutes of Brain Science, Fudan University, Shanghai 200032, China

## Abstract

**Background:**

Major-histocompatibility-complex class I-related chain A (MICA) antigens are the ligands of NKG2D, which is an activating or coactivating receptor expressed on human NK cells and CD8^+^T cells. We sought to determine whether MICA expression in human corneal epithelium (HCE) could affect the cytotoxicity mediated by NK cells or CD8^+^T cells.

**Methods:**

Cell cultures of HCE were harvested from human donor eyes. Flow cytometric analysis and ELISA was performed to determine the levels of MICA expression on HCE. Then, HCE was transfected with a lentivirus vector expressing MICA and GFP. Flow cytometric analysis, RT-PCR, western blot and ELISA were performed to check the levels of MICA expression. For cytotoxicity testing, allogeneic NK cells and CD8^+^T cells were isolated from peripheral blood mononuclear cells of healthy volunteers by magnetic cell sorting. The cytolytic activity of NK cells and CD8^+^T cells was assessed against MICA-transfected HCE (NK cells: E:T ratio = 3:1; CD8^+^T cells: E:T ratio = 10:1) using the nonradioactive cytotoxicity detection kit lactate deshydrogenase.

**Results:**

Surface expression of MICA on corneal epithelium was identified at a low level. A cell line of stable human MICA-transfected corneal epithelium was successfully established. Heightened expression of MICA on HCE was found to promote the cytotoxicity mediated by NK cells or CD8^+^T cells, which could be blocked by an anti-MICA antibody.

**Conclusion:**

MICA molecules may contribute to cytotoxic responses mediated by activated immune effector cells in corneal epithelium immunity.

## Background

Major-histocompatibility-complex class I-related chain A (MICA) manifest homology with classical human leukocyte antigen molecules, yet they neither engage β2 microglobulin [1], nor bind peptides and are not expressed on normal circulating lymphocytes [2,3]. MICA is transcribed in keratinocytes, endothelial cells, fibroblasts, monocytes, epithelial cell lines and reputedly in most epithelial tissues [4]. MICA engages the activating natural killer cell receptor NKG2D, which is found on many immune effector cells such as NK cells, CD8^+^T cells, γδ^+^T cells and part of CD4^+^T cells, and elicits a very powerful immune response [4-6]. Previous studies have revealed that MICA expression would appear to be considerably up-regulated in transformed cells of various types, particularly in those of an epithelial origin [7]. This has led to the concept that MICA is probably a marker of stress in the epithelia, which is consistent with the evidence for heat- and viral-induced up-regulation of MICA expression [8,9].

Thus far, MICA has been proposed to participate in the rejection process of solid organ transplantation, as well as the invasion and immune surveillance of tumors and viruses [10-14]. Definitive evidence is still lacking as to whether these mechanisms can also be applied to corneal epithelium immunity. In our previous study, we found low levels of surface MICA expression in corneal epithelium. The proinflammatory cytokine, interferon-gamma (IFN-γ) promoted surface MICA expression in the corneal epithelium and increased soluble MICA levels in a dose-dependent manner. IFN-γ also enhanced NK cell-mediated cytotoxicity against the corneal epithelium. Anti-MICA antibodies could further block this process. These findings might represent a possible mechanism of immune-mediated damage in conditions of corneal stress. However, even under the IFN-γ treatment at 1000 U/ml, the surface MICA positive rate of corneal epithelium was still modest. [15] In this study, we detected levels of MICA expression on cultured corneal epithelium first in vitro. Then, we established stable human MICA-transfected corneal epithelium with a lentivirus vector to determine the susceptibility of MICA-transfected human corneal epithelium to the killing mediated by NK cells or CD8^+^T cells.

## Methods

### Cell lines

This study was approved by the Ethics Committee of Shanghai Eye, Ear, Nose and Throat Hospital and adhered to the tenets of the Declaration of Helsinki. Corneal epithelium was obtained from adult eyes provided by Shanghai Eye Bank, which has informed consent for all tissue samples held, and cultured as described previously [16]. Corneal epithelial cells at passage 1 were used for the experiment.

Peripheral blood mononuclear cells (PBMC) were isolated by Ficoll-Hypaque density gradient centrifugation (Amersham Pharmacia Biotech, Piscataway, NJ, USA) from heparinized venous blood obtained from normal healthy volunteer donors. Informed consent was written and was obtained from all adults after explanation of the study. Similar to previous study, NK cells and CD8^+^T cells were isolated from PBMC by magnetic cell sorting (Miltenyi Biotec, Auburn, CA) and activated by IL-2 (Chiron, North Carolina, USA; 50 units/mL for NK cells and 100 units/mL for CD8^+^T cells) [17]. Specifically, CD3^-^CD56^+^ NK cells were selected by CD56 Microbeads after the depletion of CD3-positive cells using CD3 Microbeads. CD3^+^CD8^+^T cells were enriched from CD3-positive cells by CD8 Microbeads. According to previous report, the CD3^-^CD56^+^ NK cells obtained were over 93% pure, and CD3^+^CD8^+^T cells were over 95% pure [17]. For the cytotoxicity assay, CD3^-^CD56^+^ NK cells and CD3^+^CD8^+^T cells were cultured for another 4 days with fresh IL-2.

### Immunostaining

Human corneal epithelium cells cultured in 6-well plates were fixed in 95% ethanol for 20 min and then dried at RT. After three rinses with PBS for 5 min each and pre-incubation with 1% normal rabbit serum to block nonspecific staining, the cells were then incubated with anti-cytokeratin 12 antibody (1:100) from Santa Cruz Biotechnology (Santa Cruz, CA) for 1 h. After three washes with PBS for 5 min each, cells were incubated with a FITC-conjugated secondary antibody (1:100; Sigma-Aldrich, St. Louis, MO) for 45 min. Cells were then washed three additional times and counterstained with Hoechst 33342 (10 g/mL). Mounted with a Mowiol 4-88 medium (Sigma-Aldrich, St. Louis, MO), human corneal epithelium cells were analyzed with a fluorescence microscope.

### Transfection of human corneal epithelial cells with MICA gene

To generate constructs expressing cell-surface MICA, full-length cDNA encoding MICA isoform 1 (NM_000247) was amplified from the 293 T cell line (R&D Systems, Minneapolis, MN) using the following primers by reverse transcription (RT) PCR: forward: 5'-AAAGGATCCATGGGGCTGGGCCCGGTC-3' and reverse: 5'-AAAACGCGTGGCGCCCTCAGTGGAGCCA-3'. The sequence of the PCR-cloned MICA was confirmed by sequencing. We created a MICA-expressing lentivirus by introducing the MICA coding sequence between the BamHI site and MluI of a PWPI1-IRES-GFP lentiviral construct (plasmid 12254, Addgene, Cambridge, MA). The 293 T cells were transfected with lentivirus particles for MICA lentivirus collection. In brief, both 8 μg empty vector plasmid DNA and 8 μg MICA-IRES-GFP plasmid were mixed individually with 6 μg psPAX2 plasmid (plasmid 12260, Addgene, Cambridge, MA) and 2 μg pMD plasmid (plasmid 12259, Addgene, Cambridge, MA) and diluted to 1 mL reduced-serum medium (OptiMem; Invitrogen-Gibco). The two were then mixed and kept at room temperature for 30 min to allow complexing of the DNA with the transfection reagent. The mixture was added slowly to the 293 T cells, which had been cultured on 10-cm culture dishes in DMEM containing 10% heat-inactivated FBS. After a 6 h incubation period, culture supernatant was removed and an additional 15 mL DMEM containing 10% heat-inactivated FBS was added. The medium containing the virus was collected from days 2 to 6 after transfection, and purified by 0.45 μm filter after being spun at 4000 rpm for 10 min in a centrifuge (GPK; Beckman, Fullerton, CA). Then, we treated corneal epithelial cells with the empty virus (IRES-GFP) and the MICA-IRES-GFP lentivirus. First-passage human corneal epithelial cells were used for the infection. The cells were seeded on a 6-well culture plate at 2 × 10^5^ cells/well in KSFM. When the cells grew to about 60% confluence, the medium was changed to 2 mL defined K-SFM containing 8 μg/mL polybrene (Millipore, Billerica, MA). The concentrated virus solution (3 mL) containing the empty vector or the MICA-expressing lentivirus, was added to the well to transduced cells for 24 h and then replaced by fresh defined K-SFM for additional 48 h.

### Antibodies

Primary. Anti-human MICA monoclonal antibodies (R&D Systems, Minneapolis, MN), anti-MHC class I (eBiosciences, San Diego, CA), isotype control mouse IgG (BD Biosciences, Mountain View, CA) and mouse anti-human keratin 3/12 (Abcam, Cambridge, UK) Abs were purchased.

Secondary. Affinity-purified second Abs and species-absorbed conjugates for dual-labeling were purchased from Sigma-Aldrich (Saint Louis, Missouri, USA).

### Flow cytometry

MICA expression in corneal epithelial cells was analyzed by flow cytometry as previous described. [17] Anti-human MICA antibodies at the appropriate concentrations were added to 1 × 10^5^ cells for 30 min at 4°C, washed twice with cold phosphate-buffered saline, and stained with secondary antibodies. Each sample was analyzed with the flow cytometer (Beckman Coulter, Inc., Fullerton, CA).

### RT-PCR

Total RNA was isolated with Trizol (Invitrogen) and reverse transcription-PCR (RT-PCR) was done with the RT-PCR system (TAKARA Biotechnology, China) according to the instructions of the manufacturers. Amplified cDNAs were then performed for PCR with the following specific primers: MICA, forward 5'-ATCTTTGAGCCACGACAC-3' and reverse 5'-CTTCTTACAACAACGGACATA-3'. GAPDH, forward 5’-TTTTGCGTCGCCAGGTGA AGAC-3’ and reverse 5’- TCTGAGCGATGTGGCTCGGCT-3’. The GAPDH was detected as control. The RT-PCR band densities were determined using AlphaEase FC Software (AlphaInnotech, Inc.).

### Western blot

MICA-transfected human corneal epithelium cells were lysed using Cell Lytic M (Sigma-Aldrich). Cell lysates were analyzed by western blot using standard protocols, and an anti-MICA antibody were used as the primary antibody. An FITC-conjugated secondary antibody was added, and specific bands were visualized using the Odyssey infrared imaging system (LI-COR Biosciences, Nebraska, USA). The western blot band densities were determined using AlphaEase FC Software (AlphaInnotech, Inc.).

### Cytotoxicity assay

The cytolytic activity of CD3^-^CD56^+^ NK cells and CD3^+^CD8^+^T cells was assessed against human corneal epithelium using the nonradioactive cytotoxicity detection kit lactate deshydrogenase (LDH, Roche Biochemicals, USA) according to the manufacturer's instructions. In brief, effector cells were mixed with 1 × 10^5^ target cells in triplicate wells of 96-well U-bottom plates for 4 h at 37°C. Effector (E) to target (T) ratios (E:T) used were 3:1 for NK cells and 10:1 for CD8^+^T cells. After this, plates were centrifuged at 250 × g for 10 min and 100 μl of the cell-free culture was collected and incubated with the reaction mixture from the kit. For blocking studies, target cells were preincubated for 30 min at 4°C with 10 μg/ml of anti-MICA, anti-HLA antibody, or isotye control. After incubation for 4 h at 37°C, the assays were stopped. The LDH level was determined using tetrazolium salts in conjunction with diaphorase or alternate electron acceptors. The absorbance at 490 or 492 nm and the percent cytotoxicity for each effector were recorded: target cell ratio was calculated using the following formula: cytotoxicity = (experimental - effector spontaneous - target spontaneous)/(target maximum - target spontaneous) × 100%.

### Statistical analysis

All data are representative of experiments performed at least three times. Differences in soluble MICA and the percentage of killing among different subject groups were assessed by a nonparametric test. A 95% confidence interval (P < 0.05) was considered significant.

## Results

### Low levels of surface MICA could be detected on human cultured corneal epithelium in vitro

**Figure 1:**
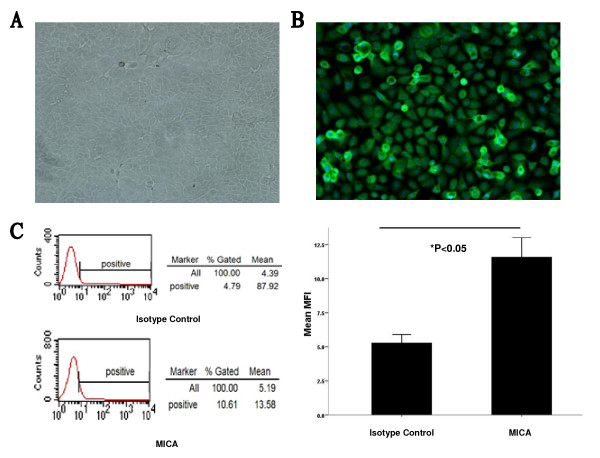
**Expression of MICA on human corneal epithelium in vitro.** (**A**) In vitro cultured human corneal epithelium was polygonal or fibroid in shape. (**B**) It was immunostained for K3/K12 (green), followed by Hochest staining (blue). (**C**) MICA-positive corneal epithelium was detected (red histogram in left figure) by flow cytometry. Results obtained from one of three samples are illustrated. Right figure shows that the MFI of the isotype control was significantly different from that of MICA.

We performed flow cytometric analyses and ELISA to determine whether human corneal epithelium cells express MICA. Previous study found that MICA detection was trypsin-sensitive [17]; thus, freshly isolated cells could not be used for flow cytometry assessment. Adult human cells were used 10 days after cell isolation, when they had recovered their physiological characteristics, reached an available number and expressed keratin-3/12, which is a biomarker for corneal epithelium (Figure 1A, 1B). In this study, surface MICA expression on corneal epithelium was identified by flow cytometry (5.7 ± 0.6%, Figure 1C), while sMICA in the supernatant could not be detected by ELISA (data not shown).

### A cell line of stable human MICA-transfected corneal epithelium was successfully established

**Figure 2:**
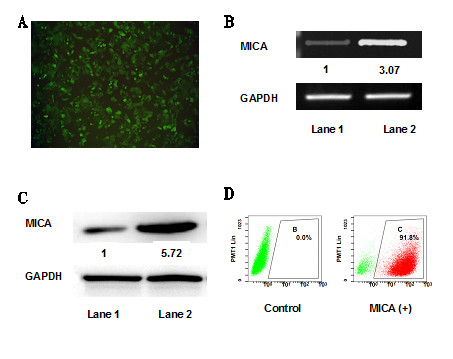
**Establishing of MICA-transfected human corneal epithelium.** MICA-transfected corneal epithelium cells were labeled with GFP (**A**). MICA RT-PCR products (**B**) and protein (**C**) from the control vector alone (Lane 1) and MICA-transfected cells (Lane 2) were both detected at 48 h after transduction. RT-PCR and western-blot analysis both show an increase in MICA expression in the transduced cells compared to the control cells. Flow cytology showed a high MICA positive rate for these transfected cells (**D**). Original magnification: A × 40.

To attempt transfection, we exposed corneal epithelium to the empty virus and the MICA-expressing lentivirus. In both groups, human corneal epithelial cells survived and proliferated. Under microscopy, these cells showed typical epithelial cell morphology and expressed GFP (Figure 2A). RT-PCR was used to demonstrate that stably transfected cells produced mRNA coding for MICA. MICA mRNA levels were significantly higher in transfected cells compared with the control (Figure 2B). Heighted protein expression of MICA on transfected cells was validated by western blot analysis using an anti-MICA monoclonal antibody (Figure 2C). Finally, the specificity of MICA staining was confirmed by flow cytometry (Figure 2D).

### Ectopic expression of MICA enhanced the killing mediated by NK cells or CD8^+^T cells to human corneal epithelium

**Figure 3:**
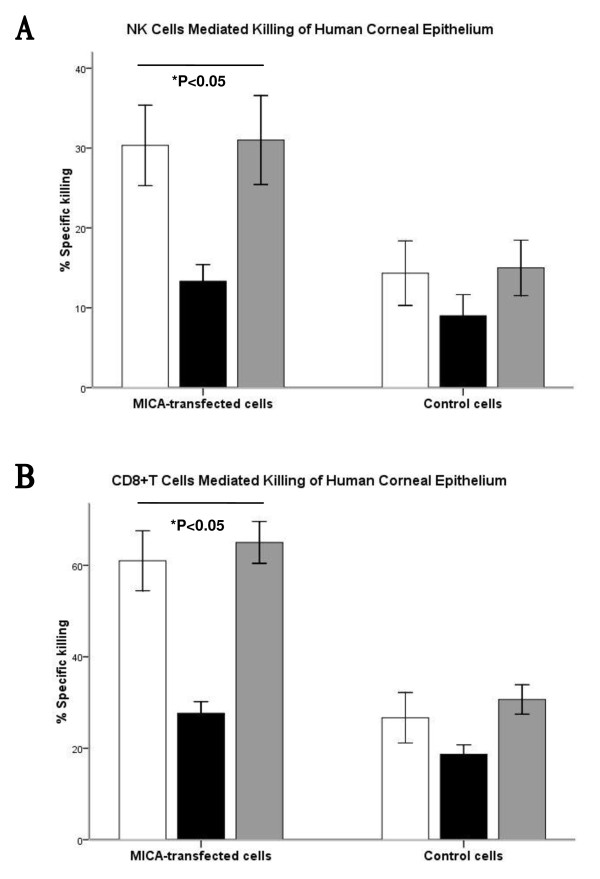
**Contribution of MICA to NK cells and CD8**^**+**^**T cells mediated killing of human corneal epithelium**. (**A**) Human MICA-transfected corneal epithelium or empty vector transfected corneal epithelium were preincubated for 4 h with either an isotype control (white bars), anti-MICA antibody (black bars) or anti-MHC class I antibody (gray bars) before being added to IL-2-activated NK cells (effector) at a 3:1 (E:T) ratio. (**B**) Human MICA-transfected corneal epithelium or empty vector transfected corneal epithelium were preincubated for 4 h with either an isotype control (white bars), anti-MICA antibody (black bars) or anti-MHC class I antibody (gray bars) before being added to allogeneic human CD8^+^T cells (effector) at a 10:1 (E:T) ratio. Nonparametric test comparing isotype and MICA antibody showed a statistical difference between groups for MICA-transfected cells (NK cells, *P* < 0.05; CD8^+^T cells, *P* < 0.05), but not for non-transfected cells. Data are presented as percentage of specific killing. Each assay was repeated three times and presented as mean ± standard error.

We evaluated the cytotoxicity mediated by allogeneic NK cells and CD8^+^T cells to MICA-transfected human corneal epithelium. IL-2-activated NK cells and CD8^+^T cells purified from allogeneic PBMC were used as effector cells in killing assays, respectively. Empty vector transfected corneal epithelium was used as the target cells control. Corneal epithelium was preincubated with an isotype control, anti-MICA antibody or anti MHC-I antibody for 1 h before being added to effector cells (NK cells: E:T ratio = 3:1; CD8^+^T cells: E:T ratio = 10:1). As illustrated in Figure 3, the specific killing was significantly inhibited only in the transfected cell group preincubated with anti-MICA. Blocking MHC class I molecules did not affect the killing, confirming that the MICA blocking was not caused by steric hindrance. Our observations demonstrated that ectopic expression of MICA on human corneal epithelium could contribute to its susceptibility to the killing mediated by NK cells or CD8^+^T cells.

## Discussion

The roles MICA molecules may play in virus infection and tumor immunology have been highlighted [4]. In this study, we determined for the first time whether MICA expression in human corneal epithelium could affect the cytotoxicity mediated by NK cells or CD8 + T cells. The flow cytometry analysis revealed low levels of expression of surface MICA on cultured corneal epithelium. Therefore, in order to evaluate the possible mechanism of the involvement of MICA expression, we established stable human MICA-transfected corneal epithelium with a lentivirus vector expressing GFP. Our data showed a higher susceptibility of MICA-transfected human corneal epithelium to the cytoxicity mediated by NK cells and CD8^+^T cells, indicating that MICA may contribute to corneal immunity.

MICA is frequently expressed in many, but not all, lung, breast, kidney, ovarian, prostate, gastric, and colon carcinomas and melanomas [7,18]. The physiological reasons are unknown, but they could be related to local stress-inducing conditions such as tumor cell proliferation, hypoxia, and hyperglycemia. The exact mechanism of regulating MICA expression remains to be fully explored. Oxidative stress has been shown to increase MICA gene expression in a colon carcinoma cell line [19]. Intestinal epithelial expression of MICA may also be inducible by bacteria [20]. In the current study, we found that corneal epithelium derived from normal human subjects expressed low levels of surface MICA by flow cytometry. We postulate that this expression pattern of surface MICA on corneal epithelium may, similarly to the low or undetectable MHC class I molecule expression, serve as a protective mechanism from immune-mediated injury [21].

However, the use of these human corneal epithelial cells for the cytotoxicity assay is hampered by their very limited MICA expression, which creates the need for an up-regulated expression or “transfected” cell line. Previous studies have shown that human corneal epithelial cells have been successfully immortalized by transformation with viral oncoproteins including the SV40 large T antigen and HPV16-E6/E7, which may be genomically unstable and display cellular properties that differ from their normal counterparts, leading to dedifferentiation, resistance to lytic and/or apoptotic cell death and therefore to variations in phenotype and functional assays [22-24]. Recently, human cell lines transfected with pWPI plasmid were found to exhibit genetic stability and were used as an ideal viral vector [25,26]. In our study, we demonstrated that HCE was successfully transfected with MICA and GFP. As shown in Figure 2, MICA mRNA was up-regulated in MICA-expressing HCE and induced more protein expression compared with control cells. Consistently, fluorescence-activated cell sorting (FACS) analysis of MICA expression revealed a higher positive rate of MICA-positive population in the experimental cell line, where > 90% of the population was MICA (+) after sorting. Together, these results suggested that this MICA-expressing cell line was available for the further cytotoxicity assay.

Several studies have revealed that rejected corneal grafts or other alter corneal disorders become infiltrated with CD4^+^T cells, macrophages, CD8^+^T cells, natural killer (NK) cells and neutrophils [27,28]. Whereas only activated and memory mouse CD8^+^T cells express NKG2D, all human NK cells and CD8^+^T cells express this receptor[29]. These two activated cell types have previously been shown to kill corneal epithelium in vitro[27,30]. We provided evidence for the first time that allogeneic NK cells and CD8^+^T cells could significantly kill MICA-transfected human corneal epithelium compared to control cells. These results are in agreement with data reported by previous studies. Suárez-álvarez et al. found that the killing activity of NKL cells was higher in the C1R-MICA transfectants than the control cell line [12]. Saikali et al. observed that disruption of the MICA-NKG2D interaction using blocking antibodies significantly inhibited killing of primary human oligodendrocytes mediated by activated human NK cells and allo-reactive CD8^+^T cells. [17] Collectively, these findings highlight the importance of MICA as a possible proinflammatory factor in corneal immunity by activating the cytotoxic capacity of alloreactive cells. Notably, because we were unable to completely arrest the killing with an anti-MICA antibody, it is possible that additional activating receptors could play a role in the observed cytotoxicity.

## Conclusion

We determined the MICA expression on human corneal epithelium in vitro for the first time and demonstrated that it could enhance the killing mediated by NK cells or CD8^+^T cells by using a MICA-transfected HCE line. In this regard, cell surface expression of MICA should be tightly controlled in order to avoid attack against autologous healthy cells, which are crucial in maintaining immune privilege in the cornea. It would be of great interest to investigate the MICA expression in altered corneal disorders, such as herpes simplex keratitis and rejected allograft; such research would be crucial in exploring a novel mechanism to the previously observed MHC restricted and non-restricted immune-mediated cytotoxicity directed at these abnormal cells.

## Competing interests

The authors declare no conflict of interest.

## Authors’ contributions

JH carried out the cell culture, participated in the flow cytometric analysis, RT-PCR, statistical analysis and drafted the manuscript. TQ carried out the immunoassays, western blot and ELISA. GL carried out the transfection. XY, JC and QL participated in the cytotoxicity testing. XS and JX designed the study and obtained funding. All authors read and approved the final manuscript.

## Authors’ information

JH and TQ are the co-first authors. Department of Ophthalmology, Eye, Ear, Nose, and Throat Hospital, School of Shanghai Medicine, Fudan University, 83 Fenyang Road, Shanghai 200031, China.

## Pre-publication history

The pre-publication history for this paper can be accessed here:

http://www.biomedcentral.com/1471-2415/12/6/prepub
